# Vascular endothelial cell specification in health and disease

**DOI:** 10.1007/s10456-021-09785-7

**Published:** 2021-04-12

**Authors:** Corina Marziano, Gael Genet, Karen K. Hirschi

**Affiliations:** 1 Department of Cell Biology, University of Virginia School of Medicine, Charlottesville, VA 22908 USA; 2 Cardiovascular Research Center, University of Virginia School of Medicine, Charlottesville, VA 22908 USA; 3 Department of Medicine, Yale Cardiovascular Research Center, Yale University School of Medicine, New Haven, CT 06520 USA

**Keywords:** Endothelial cell specification, Blood vessel development, Lymphatic vessel development, Arterial-venous malformations, Lymphatic malformations

## Abstract

There are two vascular networks in mammals that coordinately function as the main supply and drainage systems of the body. The blood vasculature carries oxygen, nutrients, circulating cells, and soluble factors to and from every tissue. The lymphatic vasculature maintains interstitial fluid homeostasis, transports hematopoietic cells for immune surveillance, and absorbs fat from the gastrointestinal tract. These vascular systems consist of highly organized networks of specialized vessels including arteries, veins, capillaries, and lymphatic vessels that exhibit different structures and cellular composition enabling distinct functions. All vessels are composed of an inner layer of endothelial cells that are in direct contact with the circulating fluid; therefore, they are the first responders to circulating factors. However, endothelial cells are not homogenous; rather, they are a heterogenous population of specialized cells perfectly designed for the physiological demands of the vessel they constitute. This review provides an overview of the current knowledge of the specification of arterial, venous, capillary, and lymphatic endothelial cell identities during vascular development. We also discuss how the dysregulation of these processes can lead to vascular malformations, and therapeutic approaches that have been developed for their treatment.

## Introduction

The vascular system is comprised of both the blood and the lymphatic circulation, which function cooperatively to maintain tissue survival, growth, function, and homeostasis. Endothelial cells (ECs) line the innermost layer of all of these vessels and play an important role in sensing the circulating environment and responding to extrinsic signals. ECs exhibit a high degree of heterogeneity in gene/protein expression and structure depending on the vessel type in which they reside (i.e., arterial, venous, capillary or lymphatic), and these acquired differences enable distinct functions, as well as tissue-specific functions.

The processes of EC specification and maturation are vastly different among all of these EC types—from the first stages of specialization to the formation of tissue-specific characteristics. Impairments in any of these developmental pathways result in vascular malformations that can be lethal. This review will highlight important regulatory pathways that promote both blood and lymphatic EC development and specification and describe vascular malformations that occur when they are dysregulated. We will also provide an overview of current therapies for these vascular disorders that have been enabled by our understanding of normal developmental pathways.

## Blood endothelial cell development

The blood vasculature is a closed circulatory system that consists of arterial, venous and capillary networks connected to the heart. The contractile property of the heart propels nutrient- and oxygen-rich blood to all tissues via the arterial system. Capillary networks between arteries and veins enable their distribution into tissues, as well as removal of metabolic waste products. Low-oxygen and low-nutrient blood is then circulated back to the heart and lungs via the venous system. Each vessel type is lined with ECs that not only exhibit a high degree of heterogeneity among different sections of the vascular tree, but also exhibit tissue-specific characteristics, especially at the capillary level. Some capillary ECs express a high level of tight junctions that restrict the passage of nutrients, soluble factors and cells in blood circulation into tissues, such as the brain and retina. In contrast, other capillary ECs have fenestrations that allow for extensive filtration of factors and cells in tissues such as the liver and kidney. Thus, each type of vessel within the blood circulation plays a crucial, yet distinct, role in maintaining tissue homeostasis.

ECs that line blood vessels are surrounded by mural cells [smooth muscle cells (SMCs) and pericytes] to a varying degree, depending on where they are in the circulatory network. For example, large arteries have a thick vessel wall made up of multiple layers of SMCs, whereas arterioles have few mural cells in their vessel wall. An exception to this is the smallest capillaries, which are only made up of a single layer of ECs that are, at times, surrounded by pericyte processes.

**Fig. 1:**
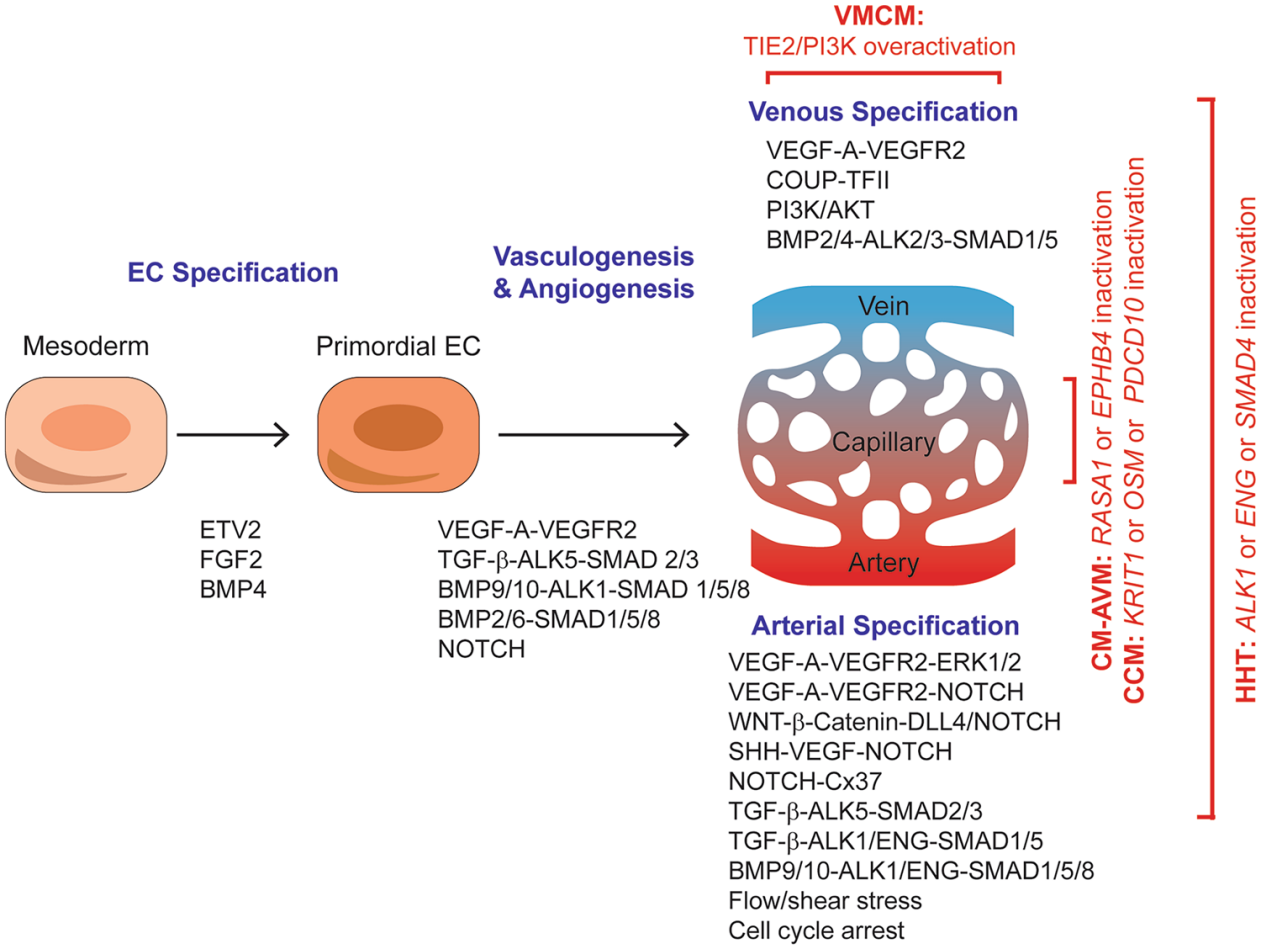
Blood EC Specification in Health and Disease. *(Black)* Primordial endothelial cells (ECs) are specified from mesoderm-derived cells and form primitive vascular plexi. Expansion and maturation of these plexi through vasculogenesis and angiogenesis forms the adult vascular network. Several developmental pathways play a significant role in initial EC specification and later in determination of arterial-venous fates. *(Red)* Changes to key signaling components in these specification pathways are associated with the development of human arteriovenous malformations, including Cutaneo-mucosal venous malformations (VMCM); capillary malformation with arteriovenous malformations (CM-AVM); cerebral cavernous malformations (CCM); and hereditary hemorrhagic telangiectasia (HHT)

Due to their close proximity to circulating factors in the blood, vascular ECs are important for the coordination of vessel responses to changes in nutrients, oxygen and other factors, such as hormones. ECs are the first vascular cells to be differentiated during development and they play a key role in the formation of their vessel wall and a complete vascular network [1, 2]. All blood ECs are thought to be derived from mesodermal progenitors, but they acquire heterogeneous characteristics as they develop and mature into arterial, venous and capillary ECs (Fig. 1). This portion of the review will focus on the signaling mechanisms regulating early blood EC differentiation and their later specification into distinct subtypes.

### Vascular endothelial cell differentiation

Vascular ECs arise from multipotent progenitors in the embryonic and extraembryonic mesoderm [3]. Newly differentiated ECs initially form a primitive vascular plexus in the extraembryonic yolk sac and later the primitive vascular plexi in embryonic tissues that give rise to the cardinal vein (CV) and dorsal aorta, as well as all forming blood vessels in developing organs. This early developmental stage that includes de novo EC differentiation and the formation of primitive vascular plexi is referred to as vasculogenesis. Vasculogenesis is initiated in the mouse at around embryonic day (E)7.0–7.5 with the expression of ETV2, a member of the E26 transformation-specific transcription factors, in mesodermal progenitors that become angioblasts, the precursors to ECs [4, 5]. This early expression of ETV2 drives the differentiation of ECs by promoting the expression of genes including vascular endothelial growth factor receptor 2 (*Vegfr2*), *Cdh5* (encodes VE-cadherin) and *Tie2* (angiopoietin receptor), which are important for the formation and maintenance of ECs [3, 6]. Other important signaling factors in the early formation of angioblasts are fibroblast growth factor 2 (FGF2) and bone morphogenic protein 4 (BMP4), which promote the acquisition of EC identity [7]. Activation of these signaling pathways is followed by the induction of vascular endothelial growth factor A (VEGF-A)-VEGFR2 signaling, which promotes EC survival, proliferation and migration, thereby expanding the primitive vascular plexus.

Some reports have suggested that arterial and venous ECs are not derived from the same population of angioblasts, but rather each develops from a distinct pool of precursors. Kohli et al. used the zebrafish model to demonstrate that the arterial and venous ECs that give rise to the major axial vessels in the fish are derived from distinct progenitor populations [8]. They show that angioblasts residing in the midline of the body give rise to the arterial ECs which develop into the dorsal aorta, while a second population of angioblasts in the lateral axis of zebrafish gives rise to venous ECs. They postulated that this difference in expression pattern was due to higher VEGF and sonic hedgehog (SHH) signaling in the midline compared to the lateral axis of the fish, which have been found to promote arterial EC development [8, 9]. Whatever the source of EC progenitors may be, the initial steps of EC differentiation, especially in the formation of the primitive vascular plexi remains largely undebated, although further investigation is needed to more fully understand the regulation of this process.

After the primitive vascular plexi are formed, blood vessels grow, expand and remodel into arterial-venous networks throughout the yolk sac and embryo proper via a process known as angiogenesis. It is important to note that not all blood vessel expansion occurs via vasculogenesis followed by angiogenesis. In some tissues, such as the brain and retina, newly differentiated ECs predominantly exhibit sprouting angiogenesis during vascular network formation, highlighting the heterogeneity in growth patterns of newly formed ECs [10–13]. Angiogenic-driven vascular expansion relies on the specification of tip cells, which are followed by several trailing stalk cells. The tip cells exhibit a migratory phenotype, while stalk cells actively proliferate to form new vessels [10, 14]; these phenotypic differences are promoted by distinct signaling mechanisms.

### Tip and stalk cell specification

As the developing embryo grows, tissue hypoxia promotes angiogenesis of new vessels to unvascularized tissues through the upregulation of VEGF-A, resulting in the induction of vascular EC sprouting [15]. VEGF-A binds to the VEGFR2 receptor on tip cells to promote cell survival and migration [16]. Loss of VEGF-A-VEGFR2 signaling axis impairs vascular development and results in early embryonic lethality [17, 18]. An important signaling factor in the maintenance of tip cell identity is the NOTCH ligand, delta-like protein 4 (DLL4) [19]. DLL4 is preferentially expressed in tip cells and is activated downstream of VEGF-A-VEGFR2 signaling. DLL4 binding to NOTCH receptors on the adjoining stalk cells promotes the expression of VEGFR1, a decoy receptor that sequesters VEGF-A, and inhibits VEGFR2 expression, thereby inhibiting migration and maintaining stalk cell identity [16, 20]. Additional pathways important for tip-stalk cell specification include TGF-β (transforming growth factor β) and BMP9/10 signaling. Binding of TGF-β and BMP9/10 to the TGF-β type-1 receptors ALK5 and ALK1, respectively, promote stalk cell identity through SMAD2/3- and SMAD1/5/8-dependent signaling [21]. Concomitantly, VEGF-A-dependent activation of Neuropilin-1 (NRP-1) in the tip cells inhibits SMAD2/3 and SMAD1/5/8 signaling ultimately repressing the stalk cell phenotype and maintaining tip cell identity [21]. Maintenance of tip and stalk cell identity is further complicated as the vascular plexus grows and matures. Induction of EC branching from already formed vessels is, in part, regulated by BMP-NOTCH signaling cross-talk, whereby BMP signaling promotes EC branching and is directly inhibited by NOTCH [22–24]. In zebrafish, BMP6 and BMP2 signaling induce EC branching through a SMAD1/5/8-dependent mechanism [22]. This is counteracted by NOTCH-dependent activation of the inhibitory SMAD6 to carefully regulate the extent of vessel branching, thereby maintaining proper vascular organization during development [22, 23].

### Arterial-venous specification

As the embryo continues to develop, the primordial ECs respond to a myriad of signals to specify into arterial or venous phenotypes. Arterial and venous ECs can be distinguished, in part, by the expression of the ligand-receptor partners E-phrinB2 and Ephrin type B receptor 4 (EPHB4) in arteries and veins, respectively [25]. EphrinB2-EPHB4 binding repels arterial and venous ECs from one another maintaining vessel identity in the developing vasculature [25]. ECs start to acquire an arterial or venous phenotype by responding to a complex signaling network which determines EC identity depending on the pathway that is dominantly activated [26]. As blood begins to flow through the developing circulatory network and ECs are exposed to mechanical shear stress, EC specification is further reinforced [27].

Multiple signaling pathways contribute to early arterial-venous specification including VEGF-A, WNT and SHH. VEGF-dependent determination of EC fate is reliant upon the relative concentration of VEGF-A, whereby high and low VEGF-A concentrations promote arterial and venous specification, respectively [28]. In arterial-fated ECs, VEGF-A-VEGFR2 drives arterial specification through activation of NOTCH- and ERK1/2-dependent signaling [29–31]. VEGF-A-VEGFR2 signaling activates the transcription factors SOX7, SOX17 and SOX18 to upregulate NOTCH-1 expression [30]. In turn, NOTCH activation and translocation into the nucleus drives the expression of arterial genes [32]. Studies in zebrafish revealed that BMP-dependent signaling potentiates NOTCH-dependent arterial specification [33]. BMP4 activation of the BMP regulatory proteins, BMPER and TWSGI, enhance NOTCH-dependent upregulation of *EphB2* [33, 34]. In parallel to NOTCH signaling, VEGF-VEGFR2 activation drives ERK1/2 (MAP kinase signaling)-dependent upregulation of arterial genes [29].

NOTCH expression is also induced through WNT-dependent signaling downstream of VEGFR2 activation [31]. Baseline WNT-β-catenin signaling induces the expression of DLL4, which promotes NOTCH activation in adjacent cells to enable arterial fate specification. Overactivation of this pathway in mice results in defects in arterial-venous specification and the formation of arteriovenous (AV) shunts [31]. SHH-dependent activation is also involved in upregulating NOTCH signaling in ECs. In zebrafish, arterial specification has been shown to be initiated by a SHH-VEGF-A-NOTCH signaling axis [9]. Overexpression of *Shh* in zebrafish leads to ectopic formation of arterial ECs, while inhibition of the SHH pathway leads to the loss of arterial identity in the developing vasculature [9]. The importance of NOTCH signaling is highlighted by the loss of arterial cell identity, accompanied by EC hyperproliferation and disrupted vascular remodeling, with the loss of NOTCH signaling [35, 36].

The initiation of blood flow through the developing vasculature further specifies arterial ECs [37]. In chick embryos, the start of blood flow in the vessels of the yolk sac upregulates arterial-specific genes including *EphB2* [37]. Arterial specification in response to flow is regulated in part by NOTCH signaling [38]. Arterial flow-mediated activation of NOTCH leads to an upregulation of mechanosensitive gap junction protein Connexin 37 (Cx37) and subsequent activation of the cell cycle inhibitor p27; p27-mediated endothelial cell cycle arrest further enables the upregulation of arterial genes [38]. The exact mechanism by which Cx37 regulates p27 and cell cycle arrest is not yet clear, and the role of endothelial cell cycle control in this process is further discussed below.

Arterial specification is inhibited to promote and maintain venous EC fate. Expression of the venous-enriched transcription factor, Nuclear Receptor Subfamily 2 Group F Member 2 (NR2F2/COUP-TFII), inhibits arterial specification through the downregulation of NOTCH signaling [39]. Similarly, ERK1/2-dependent arterial specification is antagonized by PI3K-AKT activation that drives venous EC specification [29]. PI3K-AKT-dependent inhibition of ERK1/2 promotes COUP-TFII expression in developing venous ECs [29, 40]. PI3K is activated in response to VEGF-A which, as mentioned previously, can also promote arterial specification [29, 40]. Thus, arterial-venous identity in response to VEGF-A signaling is determined by the strength of the signal and the downstream pathways activated, revealing the complexity of these developmental pathways [28]. To make matters more complex, studies in zebrafish and mice have shown that arterial ECs can be derived from venous tip cells during development [41–43]. Thus, we have much more to learn about the relationships among EC types, and the cross-talk of signaling pathways, in remodeling vascular plexi.

Another pathway that can promote divergent outcomes depending on the receptors and ligands involved is the TGF superfamily-dependent signaling. The identity of the TGF superfamily receptor activated directs whether primitive blood ECs acquire a venous or arterial phenotype. For example, in zebrafish, BMP2/4-ALK3 signaling axis activates SMAD1/5 to promote venous fate by upregulating venous-enriched genes (*Ephb4* and *Nr2f2*) [44]. Other recent studies further support the important role of BMP2-dependent signaling in promoting venous EC identity through the activation of ALK2/3 and BMP receptor II [26, 44].

In contrast, TGF-β-dependent signaling enables arterial cell fate through careful regulation of angiogenic and vascular maturation signals. The TGF superfamily type I receptors discovered to play a role in arterial development include ALK1, ALK5 and Endoglin (ENG), with ALK1 and ALK5 signaling regulating a biphasic response in ECs [45]. Mutations in ALK1 or ENG result in perturbation in arterial-venous specification and ultimate formation of AV malformations [46, 47]. *Alk1*-, *Eng*- and *Alk5-*deficient mice display severe vascular malformations resulting from the inability of the vasculature to undergo arterial-venous maturation [48–51]. In *Eng*-deficient mice, specifically, the development of AV malformations is due, in part, to the loss of ENG-dependent inhibition of COUP-TFII expression, resulting in attenuated arterial specification and increased venous EC formation [52]. Similarly, ALK1 activation has been shown to promote arterial specification during development and mice lacking *Alk1* are embryonic lethal due to severe vascular malformations [48]. Activation of ALK1 and ENG by TGF-β promotes endothelial cell migration and proliferation through SMAD1/5 signaling, enabling the expansion of developing arteries [53–55]. ALK1-ENG pro-angiogenic signaling is antagonized by ALK5 activity. TGF-β activation of ALK5-SMAD2/3 inhibits angiogenesis and promotes vessel maturation [53, 56, 57]. The interplay between ALK1 and ALK5 signaling is further complicated; that is, in vitro studies revealed that ALK5 signaling promotes ALK1 expression and is required for optimal ALK1 signaling [53]. Other studies in mice and cultured ECs argue that ALK1-dependent signaling may be more important in vessel maturation rather than angiogenesis [58–60]. The complexity of the signaling pathways and their relative contributions to proper arterial-venous specification continue to be an important area of investigation. However, it is clear that perturbation to any of these pathways severely impacts vascular development resulting in potentially lethal arterial-venous malformations [61–63].

### Cell cycle regulation of arterial-venous specification

One of the key questions in vascular development is how do similar extrinsic signals cause differential responses in developing ECs. There is growing evidence for a role of cell cycle state in determining the propensity for fate decisions during development. For example, in embryonic stem cells, different cell cycle states create distinct windows of opportunities that enable cells to differentially respond to factors that promote their differentiation toward specific lineages [64, 65]. Recent studies of vascular development similarly suggest that endothelial cell cycle state determines their propensity for arterial-venous specification [38, 39].

Although remodeling ECs are proliferative, their growth is suppressed as arterial-venous specification ensues, suggesting a role for cell cycle arrest in the acquisition of arterial-venous EC identity [38, 43, 66]. In support of this idea, our lab has recently shown that arterial shear stress activates a NOTCH-Cx37-p27 signaling axis to promote cell cycle arrest in G1 phase, which enables the upregulation of arterial genes [38]. Conversely, in venous ECs, COUP-TFII expression promotes cell cycle progression and reduces arterial EC gene expression [39]. Recent work from our group, using FUCCI cell cycle reporter mice [67], revealed that ECs in remodeling veins and arteries are in different states of G1; early G1 vs. late G1, respectively [68]. In addition, these distinct cell cycle states enable BMP vs. TGF-β signaling to upregulate venous vs. arterial genes, respectively [68]. Together, these data highlight the importance of cell cycle regulation in EC fate decisions, although much more work in needed to understand the molecular underpinnings of this process.

### Capillary Endothelial Cell Development

Connecting arterial and venous circulation is a vast network of capillaries that serve as the site of gas and nutrient exchange between tissues and the circulatory system. Developing capillaries are composed of a single layer of ECs connected by a thin basement membrane. As capillary networks mature, ECs recruit mural cell precursors via PDGF-B signaling [1, 69]. Upon contact with the capillary ECs, TGF-β is activated and promotes the differentiation of pericytes, which aid in maintaining capillary vessel integrity [1, 70–74].

There is a growing appreciation for the high degree of heterogeneity among EC phenotypes, not only in arteries and veins, but also in capillaries. The structure of capillaries is organ-specific, with morphologies that are perfectly suited for the needs of the tissue [10]. Although the structural heterogeneity among capillary ECs is well described, the transcriptional regulation required to promote these specialized characteristics remains unclear. In early embryonic development, capillaries form the primitive vascular network in the extra- and intra-embryonic tissues [75]. These primitive vascular plexi later remodel and mature into circulatory networks containing arteries, veins and mature capillaries. During the maturation process, some of the primitive capillary networks are pruned away, while new capillaries are formed through angiogenic sprouting of existing blood vessels [75]. Angiogenic sprouting is driven by the migration of endothelial tip cells followed by the proliferative stalk cells to form new capillary tubes, which will migrate until they reach other capillaries and coalesce to form new vessels [16].

As mentioned above, ECs formed from venous tip cells migrate through the remodeling capillary plexi to contribute to forming arterial vessels [41]. Due, in part, to this venous to artery migration, brain capillary ECs share gene expression patterns of both venous and arterial ECs [76]. An analysis of the developing coronary vasculature showed that ECs from the developing coronary plexus are most transcriptionally similar to adult venous and capillary ECs, suggesting that the developing adult coronary capillaries are derived from venous ECs and may only begin to express arterial markers once they make contact with arterial branches [39]. Other recent single-cell RNA sequencing analysis of murine brain capillary ECs also reveals a continuum of gene expression among capillary, arterial and venous ECs [76]. However, distinct capillary EC markers and the specification events that determine capillary EC fate remain to be elucidated.

## Arterial-venous malformations

Dysregulation of EC specification leads to vascular malformations, which can be debilitating and even fatal. There are different types of vascular malformations, depending on which process in arterial-capillary-venous development is disrupted (Fig. 1, Table 1). In this section, we provide an overview of different types of blood vascular malformations, what is known about their underlying genetic defects, and what treatments are currently available for affected patients. We also propose new research pathways linking endothelial cell cycle state and fate in the development of vascular malformations.

**Table 1:** Summary of genes, signaling pathways, and endothelial cell function modified in vascular malformations and associated animal models and treatments

Malformation	Genes	Signaling pathway	Function /Pathway	Animal models	MOlecules
**Arterio-venous anomalies**					
Hereditary Hemorrhagic Telangiectasia (HHT)	Loss of function *ENG, ALK1, SMAD4, GDF2*	Inhibition of BMP9/10 signaling (SMAD 1/5/8) Increased VEGF/ANGTP2 signaling	EC hyperproliferation Loss of vascular specification Altered FSS Hypoxia	*Eng*^ECiKO^ *Alk1*^ECiKO^ *SMAD4*^ECiKO^ BMP9/10 antibodies	Bevacizumab* Nintedanib† Pazopanib† Tacrolimus* Sirolimus*
**Venous anomalies**					
Venous malformations (VM)	Gain of function *TIE2/TEK*	Increased Pi3K/AKT signaling Decreased FOXO1 activity	EC hyperproliferation Decreased PDGF-B Decreased mural cell coverage	*Pik3ca*^H1047R^	Sirolimus† Alpelisib†
**Capillaries anomalies**					
Capillary malformation-arteriovenous malformation (CM-AVM)	Loss of function *RASA1, EPHB4*	Increased Ras/MAPK signaling	_	_	_
Cerebral cavernous malformations (CCM)	Loss of function *CCM1, CCM2, CCM3*	Increased TGFβ/BMP signaling	Endothelial-to-mesenchymal transition (EndomT) Impaired EC-EC junction Impaired EC migration	*Ccm1*^ECiKO^ *Ccm2*^ECiKO^ *Ccm3*^ECiKO^	Simvastatin† Fasudil† Exisulib† Sorafenib†
**Lymphatic anomalies**					
Type I lymphedema (early onset)	Loss of function *FLT4*	Decreased VEGFR3 signaling	Impaired lymph-vessel formation / organization	*Chy*	Sirolimus* Anti-VEGF-C-based therapy†
	Loss of function *PTPN14*	Increased VEGFR3 signaling	_	_	
	Loss of function *GATA2*	decreased *PROX1* and F*OXC2* expression	_	_	
Type II lymphedema (late-onset)	Loss of function FOXC*2*	impaired VEGF-C / VEGFR3 induced response	LEC hyperproliferation Impaired lymphatic valve development -Cx37 downregulation	*Foxc2* ^ECiKO^	
	Loss of function *SOX18*	*Flt4* expression dysregulation	Prox1 inhibition	*Ragged*	

^*^Molecule used in clinic

^†^Molecule used in preclinical studies

### Hereditary Hemorrhagic Telangiectasia

Hereditary Hemorrhagic Telangiectasia (HHT, OMIM 187300), also known as Osler-Rendu-Weber syndrome, is a genetic vascular disorder affecting 1 in 5,000 individuals worldwide [77]. This disease is characterized by multiple vascular defects including epistaxis, telangiectasias and AV shunts in various organs including brain, lungs and intestines, which are prone to rupture, causing life-threatening hemorrhage [77].

The HHT-causing mutations affect genes encoding different components of the BMP9/10 pathway; namely, *ENG* encoding the membrane glycoprotein ENDOGLIN in HHT1, *ACVRL1* encoding the membrane receptor ALK1 in HHT2, *SMAD4* encoding the intracellular signaling molecule SMAD4 in Juvenile polyposis (JP)-HHT*,* and *GDF2* encoding BMP9 ligand in HHT5 [78–82]. To date, all identified mutations result in haploinsufficiency of these genes. In ECs, decreased activity of the BMP9/10 signaling leads to over-activation of the pro-angiogenic factors VEGF-A and angiopoietin-2 (ANGPT-2), triggering EC hyperproliferation, as well as alterations in their permeability and migration, ultimately leading to vascular malformations [78, 83–85].

Mouse models of HHT have provided significant insights regarding the functions of the BMP9/10 signaling pathway defects in the development of the vascular anomalies related to HHT. Heterozygous mutations of *Eng* or *Alk1* genes give rise to vascular lesions forming at low frequencies and later in life, making them inconvenient models for further study [86, 87]. Constitutive inactivation of *Eng*, *Alk1* and *Smad4* genes in mice leads to embryonic lethality due to multiple cardiovascular defects, again rendering the study of molecular mechanisms difficult [58, 88, 89]. However, postnatal tamoxifen-inducible, EC-specific homozygous deletion of any of these genes induces HHT-like vascular malformations, including excessive angiogenesis, enlarged veins and AV shunts in the neonatal retinal vascularization model [84, 85, 90, 91].

The dysregulation of arterial-venous identity of ECs forming AV shunts has been shown in these different experimental models. The inducible EC-specific deletion of *Eng*, *Alk1* or *Smad4* leads to EC hyperproliferation, which is associated with downregulation of several arterial-enriched genes, such as *Efnb2*, *Jag1* and *Unc5b*, as well as upregulation of venous-enriched genes *Ephb4* and *Nrp2* [84, 85, 90]. These modifications are also accompanied by increased SMC coverage, or muscularization, of veins and AV shunts, while arteries undergo loss of SMC coverage or de-muscularization.

The molecular mechanism(s) leading to the dysregulation of endothelial identity in AV malformations (AVM) has not been unraveled. However, the modifications of the environment surrounding the ECs induced by AVM, such as defective flow shear stress or impaired nutrient and oxygen supply, could play an important role in this regulation. In fact, impaired-systemic blood flow in mice produces defects in arterial-venous specification and induces AVM during embryonic development [92]. In the context of HHT, fluid shear stress has been shown to potentiate BMP9 signaling through ALK1 and SMAD4 and, thus, enabling the repression of EC proliferation, as well as pro-angiogenic signals [85, 93]. Furthermore, as mentioned above, it has been shown that arterial shear stress, via the NOTCH-Cx37-p27 axis, regulates the cell cycle state of ECs and enables their arterial specification [38]. Nevertheless, the demonstration that fluid shear alterations or endothelial cell cycle state modifications in AVM could be responsible for the loss of EC identity are lacking and represent important research pathways to identify new targets for HHT treatment.

To date, the therapeutic options available for HHT patients are intended to reduce the symptoms of the disease (epistaxis). However, new clinical and preclinical studies are emerging to counter-balance the pro-angiogenic axis over-activated in HHT and, ultimately, to correct telangiectasias and AV shunts into a normal vasculature. The humanized monoclonal anti-VEGF-A antibody, bevacizumab [94], currently in phase III of clinical trial, has significantly reduced bleedings and liver and cardiac anomalies in HHT1, HHT2 and JP-HHT patients [95]. Anti-angiogenic therapies using tyrosine kinase inhibitors targeting VEGF-A signaling such as Nintedanib [96] or Pazopanib [97] have shown encouraging results in treatment of HHT-related bleeding and are now candidates for clinical trials [78]. In mouse models of HHT (*Smad4*ECiKO and *Alk1*ECiKO), anti-ANGPT-2 antibodies and PI3K inhibitors have demonstrated robust effects on correcting AVM [83, 84]. Finally, instead of targeting the pro-angiogenic signal, recent therapeutic strategies attempt to restore the BMP9-ALK1-SMAD signaling axis which is defective in HHT. In support of this approach, it has shown that the re-expression of *Alk1* gene in *Alk1-*deficient mice restores BMP9 signaling in ECs and rescues vascular malformations linked to HHT [98]. In a preclinical model (BMP9/10 immunodepletion), Tacrolimus and Sirolimus have proven their efficiency in resolving AVM by promoting the re-activation of the BMP9 signaling pathway [99].

## Venous malformations

Venous anomalies are among the most frequent cause of mortality due to vascular defects with an incidence estimated at 1/5,000 – 1/10,000 [100]. The two major categories of venous anomalies are cutaneo-mucosal venous malformations (VMCM, OMIM 600195), representing 95% of cases, and glomuvenous malformations (GVM, OMIM 138000), accounting for the majority of the remaining 5%. Contrary to VMCM, which are linked to EC dysfunction, GVM are due to impaired vascular SMC differentiation and will not be the focus of this review [101].

VMCM are congenital lesions of distorted venous-like vessels. These lesions are soft, compressible, light-to-dark blue, mainly located in the skin and mucosa, but can infiltrate underlying tissues, muscles and joints [102]. Histologically, VMCM are characterized by enlarged vessels, irregular lumens, monolayer of ECs and irregular SMC coverage [103]. Chronic activation of the EC TIE2-PI3K signaling pathway is considered the major cause of VMCM [104].

Genetic and molecular studies have shown that VMCM are caused by gain-of-function mutations in the EC-specific tyrosine kinase receptor *TIE2* (*TEK)* gene [103] or in *PIK3CA* gene, encoding the p110α catalytic subunit of PI3K [105]. Even if the downstream mechanism of TIE2/PI3K over-activation is not completely understood, several cellular and molecular dysregulations have been identified in VMVC pathogenesis, including defective EC-SMC interactions and venous identity [106–108]. In a mouse model that mimics human VMCM, the endothelial expression of *Pik3ca*^*H1047R*^, a constitutively active mutant of the p110α, results in EC hyperproliferation, reduction in mural cell coverage of blood vessels, and modification in arterial-venous identity [106]. The venous markers COUP-TFII and EPHB4, and the arterial marker Ephrin-B2 are reduced in EC-*Pik3ca*^*H1047R*^ postnatal retinas and lungs compared to those in *Pik3ca*^*WT*^ mice. However, in VMCM patient biopsies, EC proliferation is not a noticeable characteristic [103, 109]. These divergences could be due to the different models and time points of the studies. As already suggested, VMCM could have an initial phase of EC proliferation, where they are more responsive to proliferative cues, followed by a more quiescent state in established lesions [104].

A recurrent hallmark of VMCM is defective SMC coverage in venous lesions [103, 105]. As a consequence of TIE2/PI3K over-activation, the transcription factor FOXO1 and its target gene encoding for PDGF-B, a SMC chemo-attractant, have been shown to be negatively regulated in *Pik3ca*^*H1047R*^ mice and biopsies from TIE2 mutation-positive VMCM lesions [106, 107]. To date, it is not known whether decreased PDGF-B levels leads to defective SMC coverage in VMCM lesions. However, understanding the consequences of impaired EC-SMC interactions on EC identity in venous malformations could enhance our understanding of how these lesions are formed. Specification of arterial and venous ECs occurs in conjunction with suppression of EC cycle progression [7, 38, 39]. FOXO1 activation plays a role in the regulation of cell cycle progression by promoting cell cycle arrest via the cyclin-dependent kinase inhibitor p27kip1 [110, 111]. Nevertheless, the dysregulation of endothelial cell cycle regulators, such as p27, and its impact on venous identity in VMCM induced by over-activation of PI3K-AKT-FOXO1 axis still need to be investigated.

There is currently no available treatment for VMCM patients. However, in a few preclinical studies, specific inhibitors of TIE2 or PIK3CA over-activation have been tested to prevent or revert VMCM. TIE2 kinase inhibitor or PIK3CA inhibitor (Alpelisib) have demonstrated very modest effects on treating VMCM [112, 113]. The most advanced potential pharmacotherapy for VMCM currently is Sirolimus (also known as Rapamycin). This compound inhibits TIE2- and PIK3CA-mutated venous malformations in vitro and in vivo and, in mouse models, it diminishes lesion growth, normalizes SMC coverage and decreases EC proliferation [106, 112].

## Capillary malformations

Capillary malformations (CM, OMIM 163000), also called “port wine stains”, are the most common type of cutaneous vascular malformation, affecting 0.3% to 0.5% of the population [114]. CM consist of dilated capillary-like vessels and lesions that are sporadically flat, red to purple in color, and found most frequently in the head and neck [115]. CM are divided into two categories: CM with AV malformations (CM-AVM, OMIM 608354); and cerebral cavernous malformations (CCM, OMIM 116860) [100].

### Capillary malformations with arterial-venous malformations

CM-AVM is an autosomal dominant disorder caused by heterozygous inactivating mutations in the *RASA1* (RAS p21 protein activator 1) gene (CM-AVM1) characterized by the presence of capillary malformations, arteriovenous malformations and fistulas, and occasionally vascular overgrowth [116, 117]. *RASA1* encodes the RAS-GTPase-activating protein, p120Ras-GAP, that negatively regulates the RAS/MAPK signaling pathway [117, 118]. Among different functions, Ras is an activator of VEGF-A-mediated angiogenesis by promoting the phosphorylation and stabilization of the hypoxia-inducible factor-1 alpha (HIF-1α) transcription factor, which upregulates VEGF-A [119, 120]. As a negative modulator of RAS activity, p120Ras-GAP plays a role in balancing EC signaling by downregulating proliferation, and potentially migration and polarity [121, 122].

There is no ideal vertebrate genetic model of CM-AVM currently available [123, 124]. *Rasa1* heterozygous mutant mice are viable, but the homozygous deletion of *Rasa1* induces embryonic lethality at E10.5 due to defective vascular development [124]. Overexpression of microRNA-132, a negative regulator of p120Ras-GAP protein expression, promotes neovascularization in a murine tumor model, whereas anti-microRNA-132 inhibits this effect and prevents pathological retinal angiogenesis [121, 125]. CM-like vascular lesions have been reported in those models, but not CM-AVM. Nevertheless, a study using Morpholinos against *Rasa1* in zebrafish showed that reduced *Rasa1* gene expression leads to impaired circulation and arterial-venous misconnections [123]. Interestingly, this study provides a molecular connection between RASA1 and EPHB4, which is well known for its function in venous specification during embryonic development and is considered a venous marker in the adult vasculature [126, 127]. However, whether dysregulation of EC identity contributes to capillary malformations is still unknown. *EPHB4* variants were also reported to cause capillary malformation–arteriovenous malformation 2 (CM-AVM2) [128]. CM-AVM2 mimics RASA1-related CM-AVM1 and HHT and could therefore be considered part of the clinical spectrum of HHT and other vascular malformation syndromes [129].

### Cerebral cavernous malformations

CCM occur in approximately 0.5% of the population and can be both sporadic (80%) or autosomal dominant inherited (20%) [130]. The lesions are mostly localized in the brain, but also in the spinal cord and retina [131]. Histologically, CCM consist of dilated vessels, known as cavernomas, that are often assembled into clusters (i.e., mulberry lesions) [130]. These abnormal vessels are formed by a defective layer of ECs that lacks tight junctions and is prone to blood leakage and vascular rupture [131]. Patients can develop symptoms such as headaches, seizures, neurological problems and cerebral hemorrhages, although some are asymptomatic [131, 132].

The inherited forms of CCM are caused by a loss of function mutation in any of the 3 autosomal genes, *CCM1/KRIT1*, *CCM2/OSM* or *CCM3/PDCD10* [130]. In sporadic forms of CCM, somatic mutations of CCM genes have been observed in cerebral cavernomas [133]. *CCM1-3* mutations in ECs are mainly responsible for the vascular defects observed in patients with CCM. Endothelial-specific deletion of *Ccm1*, *Ccm2*, or *Ccm3* genes in mice induces CCM-like vascular defects in the central nervous system [134, 135], whereas SMC- or neuro-specific mutations of *Ccm* genes do not induce cavernomas [136]. Loss of function of any of the *Ccm* genes leads to increased TGF-β/BMP signaling, EC junction defects and endothelial-to-mesenchymal transition (EndMT), which collectively contribute to cavernoma formation [134, 135, 137].

The CCM proteins interact with each other, with CCM2 acting as a linker between CCM1 and CCM3 [138]. The complex binds to VE-cadherin at EC-EC junctions, through β-CATENIN [139]. In the absence of CCM1 or CCM2, the activation of RAP1, a small GTPase known to stabilize the cortical actin cytoskeleton, is impaired [140], and CDC42 is inhibited [141]. This, in turn, promotes disorganization of EC junctions and increases vascular permeability, leading to hemorrhage [142]. Interestingly, endothelial-specific gene deletion of *Cdc42* leads to capillary and venous malformations in postnatal mouse retina, similar to CCM defects, due to impaired EC polarized migration [143].

Enhanced TGF-β/BMP signaling after deletion of any of the CCM genes is a common dysregulation causing cavernomas. Two different mechanisms have been identified as responsible for this increased signaling: higher and sustained response to TGF-β by *Ccm*-deficient ECs; and production of endogenous BMP ligands, such as BMP2 and BMP6 [134, 144, 145]. Increased TGF-β/BMP signaling, through an upregulation of the transcription factor KLF4, promotes EndMT, which contributes to the loss of EC identity in cavernomas [134, 145, 146]. EndMT is characterized by disorganization of EC junctions [134], loss of apical-basal polarity [147], and expression of stem/mesenchymal cell markers [134]. Furthermore, CCM1, via the DLL4-NOTCH pathway, promotes mRNA expression of the cell cycle inhibitors *p21* (*CIP1*) and *p27* (*KIP1*), which inhibit EC proliferation and migration in vitro [148]. Therefore, loss of function of any CCM gene could disrupt endothelial cell cycle control via inhibition of p21 or p27. Since endothelial cell cycle control, via a NOTCH-Cx37-p27 axis, is necessary for the acquisition of arterial EC cell identity, it is possible that dysregulation of this axis contributes to CCM [27, 38]. Thus, further investigation of this pathway could provide additional understanding of the molecular mechanisms leading to CCM and reveal new targets for their treatment.

No pharmacological therapy is currently available for CCM. A partial surgical resection of the vascular lesions is the only therapeutic option at present. However, this strategy is not feasible in critical regions of the brain and does not prevent lesion resurgence. The ideal therapy would be to stabilize the preexisting lesions, inhibit their progression and block the formation of new malformations [149]. Using in vivo and in vitro experimental models of CCM, several pharmacological agents have been tested in preclinical studies. Inhibitors of RhoA (Simvastatin) or ROCK (Fasudil) may be good candidates [150]. Both drugs significantly decrease chronic hemorrhage in vascular lesions in murine model of CCM1 and CCM2. However, Fasudil is more efficient than Simvastatin in improving survival and blunting the development of mature lesions [150]. Exisulib, a β-catenin signaling inhibitor, limits the formation of brain vascular cavernomas in mice with CCM3 ablation in ECs [135]. This drug is currently used clinically to treat different pathologies and may be repurposed for CCM therapy. Similarly, other compounds such as ANGPT-2 neutralizing antibodies [151], multiple kinases inhibitor (Sorafenib) [148] or TGF-β receptors inhibitors [134], have shown efficacy in reducing CCM lesions in preclinical studies, but have not been tested in clinical trials yet.

## Lymphatic endothelial cell development

The lymphatic circulation is a specialized vascular network responsible for maintaining tissue fluid homeostasis, immune cell transport and lipid absorption [152]. It is comprised of blunt-ended capillaries, collecting lymphatic vessels and lymph nodes which transport fluid, or lymph, unidirectionally from tissue capillaries into the venous circulation through the subclavian vein [152]. This unidirectional flow of lymph is aided by the presence of intraluminal lymphatic valves within the collecting lymphatic vessels [153]. These lymphatic valves, much like venous valves, help propel lymph against the force of gravity, while preventing retrograde transport. Pathologies which affect the development of the lymphatic circulation result in poor lymphatic flow and severe tissue edema [154–156]. Although the end pathologies resulting from aberrant lymphatic development are well understood, there is some debate about the processes that regulate lymphatic development, specifically the source of progenitor cells that give rise to lymphatic ECs (LECs).

LECs form the inner layer of lymphatic vessels and display a high degree of structural and functional heterogeneity throughout the lymphatic network that is evident from the beginning of development. LECs are specified shortly after blood ECs develop from the embryonic mesoderm. In 1902, Florence Sabin was the first to identify venous ECs as a source of LEC progenitors in developing lymphatic vessels in pig embryos [157]. These studies were later supported by lineage tracing of PROX1^+^ cells (master regulator of LECs described below) in mice and zebrafish, which revealed that venous-derived LEC development was conserved throughout different animal species [158–160]. As animal models and lineage-tracing techniques improved, additional sources of lymphatic progenitor cells were identified. In 2015, Stanczuk et al. identified cKit-expressing cells as a novel, non-venous source of LECs in the developing mesenteric lymphatics of the mouse embryo. We will discuss lymphatic vessel development from both progenitor sources [161].

Specification of LECs and their growth pattern differ depending on the source of LEC progenitors and the tissue in which they are developing. Most venous-derived lymphatic vessels form via a process known as lymphangiogenesis, in which newly specified LECs sprout from the endothelium of the CV in organized branches [162, 163]. Conversely, most non-venous-derived lymphatic vessels form via a process known as lymphvasculogenesis [164]. In this process, newly specified LECs form clusters of cells that coalesce into lymphatic vascular plexi before being remodeling into a mature lymphatic network [164]. Regardless of the source of progenitors, all LECs share distinct genetic markers that are unique to the lymphatic circulation.

Cellular markers and their role in LEC development have been reviewed previously [165–169]. Briefly, the unique markers that are used to identify LECs include the transmembrane O-glycoprotein podoplanin (PDPN, gp38, or T1α), the transmembrane receptors that bind vascular endothelial growth factor C (VEGF-C), vascular endothelial growth factor receptor 3 (VEGFR3 or FLT4) and NRP-2, the lymphatic vessel endothelial hyaluronan receptor 1 (LYVE1), and the transcription factors forkhead box C2 (FOXC2) and prospero homeobox 1 (PROX1). Arguably the most important of these genes is the transcription factor PROX1, whose expression commits progenitor ECs into an LEC fate [170, 171]. Embryonic loss of *Prox1* expression impairs lymphatic development [163]. Furthermore, *Prox1*-deficient mice develop severe edema in utero and are embryonic lethal, emphasizing the importance of PROX1 in LEC specification and lymphatic development [163].

Although PROX1 expression is required for the fate commitment of all LECs, their response to growth factors during lymphatic vessel formation appears to be tissue-specific [161, 164, 172]. For example, VEGF-C, the prominent VEGF isoform responsible for promoting lymphatic expansion, affects lymphatic development in different tissues at different developmental time points. That is, *Vegf-C* overexpression promotes excess lymphatic development in the respiratory tract only during embryogenesis, while promoting dermal lymphatic growth throughout development and into adulthood [173–175]. These data highlight the importance of studying lymphatic development in a tissue- and time-specific context. The following sections will review the currently available data about lymphatic endothelial progenitor cell origins and tissue-specific lymphatic vessels into which they develop.

### Venous-derived LECs

**Fig. 2:**
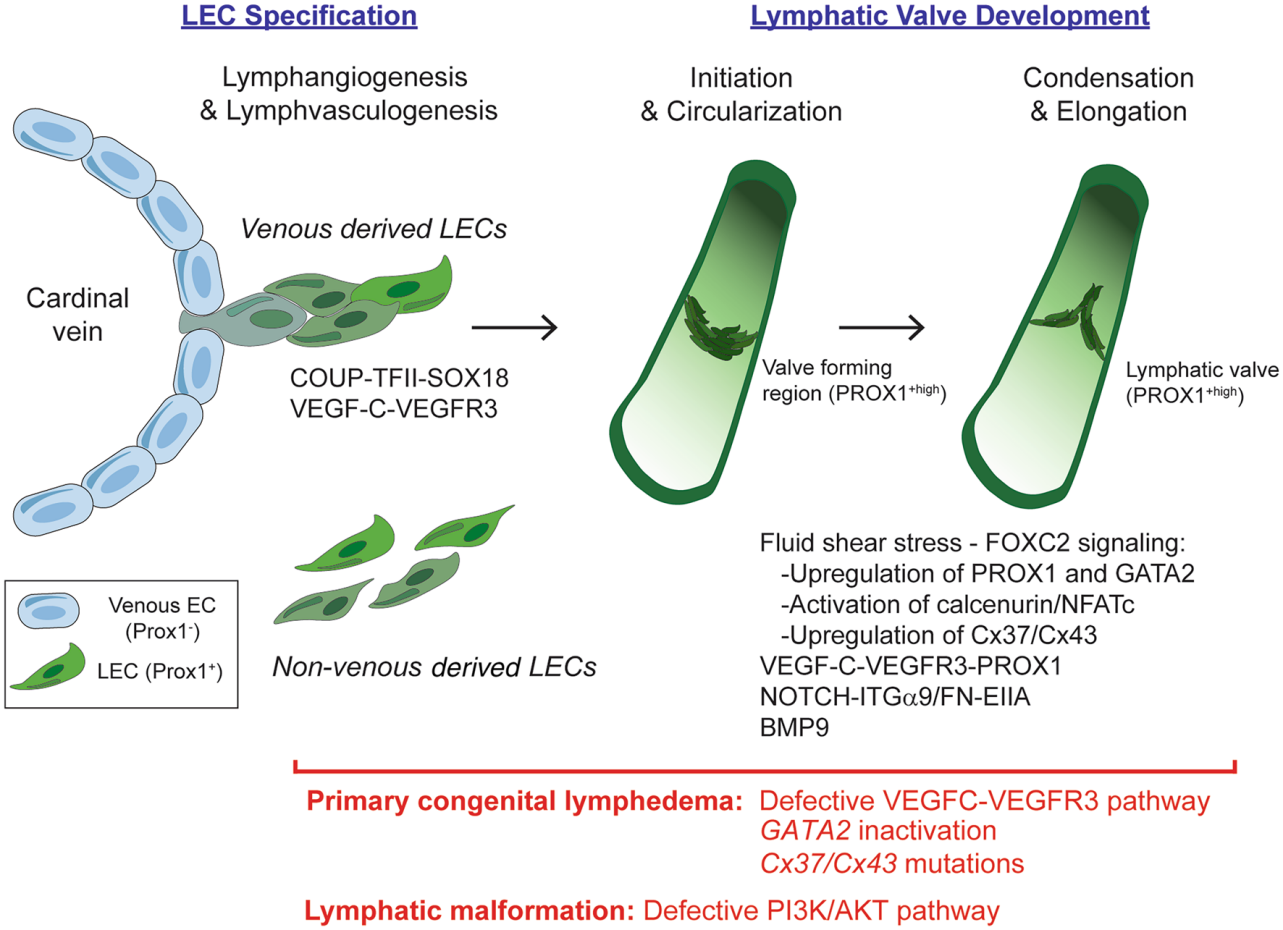
Lymphatic EC Specification and Lymphatic Valve Development. *(Black)* Lymphatic ECs (LECs) are specified from venous and non-venous progenitor cells. LECs form the embryonic lymphatic circulation through lymphangiogenesis and lymphvasculogenesis. Collecting lymphatic vessels have intraluminal valves which are formed from LECs with a high expression of PROX1 (PROX1^high^) and involves an intricate network of signaling pathways and transcription factors. (*Red*) Pathways disrupted in human lymphatic development that contribute to lymphatic malformations and lymphedema

Venous-derived LECs are specified from the venous ECs in the dorsolateral region of the embryonic CV in mice as early as E9.5 (Fig. 2). Increased expression of PROX1 in this subset of venous ECs drives LEC fate specification [158–160, 176, 177]. Increased PROX1 expression is regulated by the transcription factors partners COUP-TFII and SOX18 [178, 179]. COUP-TFII is expressed by venous ECs and, alone, does not drive PROX1 expression. However, when associated with SOX18, it promotes PROX1 expression, and LEC specification and expansion. Loss of either *Coup-tfII* or *Sox18* suppresses LEC specification in the CV [179, 180]. Unlike COUP-TFII, SOX18 expression is restricted to the dorsal region of the CV, which helps guide the polarized specification of LECs during lymphatic development. SOX18 expression is required for lymphatic specification, as evidence by the development of edema and embryonic lethality in *Sox18*-deficient mice [179]. Concomitantly, increased PROX1 expression enhances SOX18 expression, thereby promoting LEC specification and lymphatic development. This feedback mechanism assures the commitment of LECs from venous ECs, thereby promoting the formation of lymphatic vessels.

Work in the zebrafish model has identified that this LEC specification event happens through the asymmetric division of venous EC progenitors which results in the formation of one venous EC daughter cell and one LEC daughter cell [181]. The newly formed LECs retain expression of COUP-TFII and SOX18, which continue to promote PROX1 expression in the new LEC population [181]. Specified LECs begin to migrate dorsally out of the CV via lymphangiogenesis. Much like angiogenesis during blood vessel formation, lymphangiogenesis involves the sprouting of specified ECs and directionally driven migration. Migrating LECs travel dorsally as a single unit connected by VE-cadherin junctions along the embryo to form the jugular lymphatic sacs and the primordial thoracic ducts [177, 182]. The integrity of these junctions is crucial for proper lymphatic formation and prevention of edema during lymphatic vascular development [182]. Once these structures are formed, LECs begin to migrate once again forming the longitudinal thoracic vessels that will ultimately develop the cervical and thoracic dermal lymphatics and part of the cardiac lymphatic system [158, 176].

Recent reports have identified retinoic acid (RA) as an important signaling molecule regulating the polarized specification of LECs within the CV. Bowles et al*.* showed that RALDH2 expression, the RA synthesizing enzyme, is higher in the ventrolateral region of the CV, thereby maintaining venous EC identity [183]. Conversely, increased expression of CYB26B1, the enzyme responsible for degrading RA, is co-expressed in the dorsal CV coinciding with LECs expressing PROX1 in E9.5 mouse embryos [183]. In addition, LEC specification and lymphatic vascular growth is impaired in *Cyb26b1*^−/−^ mice [183]. Together, these data support the concept that carefully regulated levels of RA play an important role in the localized regional specification of LECs from venous ECs.

Spatiotemporal regulation of LEC specification from the CV may also be regulated by BMP signaling [169, 184–186]. Using BMP reporter mice, Beets et al*.* confirmed active BMP signaling in LECs and ECs from the CV in developing embryos [184]. BMP-dependent signaling can have differential effects on LEC specification depending on the identity of the ligand-receptor activated. In zebrafish, BMP2-SMAD1/5/8 signaling inhibits LEC specification. *Bmp2-*overexpressing zebrafish have impaired thoracic duct development due to attenuated LEC specification [185]. Similarly, *Bmp9*^*−/−*^ mice have dilated lymphatic vessels at E15.5, suggesting that BMP9-dependent signaling inhibits overexpansion of LECs during development [186]. In cell culture experiments, BMP9 signaling decreases PROX1 and LYVE1 expression, although this effect may be concentration-dependent [186, 187]. Taken together, BMP2/9 signaling inhibits LEC specification during early development in order to maintain localized lymphatic specification from the dorsolateral region of the CV.

After LECs have been specified in the CV, they migrate dorsally out of the vein to form the first lymphatic vessels. Guided LEC migration is predominantly controlled by VEGF-C-VEGFR3 signaling. As LECs become specified, they begin to express VEGFR3 under the transcriptional regulation of PROX1 [188]. As previously mentioned, VEGFR3 is highly enriched in LECs and its expression is used to distinguish between lymphatic and blood ECs. Increased expression of VEGFR3 at E10.5 enables LECs to respond to VEGF-C signaling to promote LEC migration out of the CV [189, 190]. In *Vegf-c*-deficient mice, PROX1^+^ LECs fail to migrate out of the CV resulting in severe edema [162]. Conversely, overexpression of *Vegf-c* results in ectopic lymphatic growth [191].

A recent report put forth the hypothesis that LEC fate is predetermined at early stages of development prior to CV formation [192]. Stone and Stainier argue that, unlike blood ECs which are derived from the lateral mesoderm, LECs are specified from the paraxial mesoderm and migrate to form the dorsal CV before finally differentiating into LECs [192]. Another group showed a similar pattern in the developing zebrafish. Nicenboim et al*.* present evidence that LECs are not derived from blood ECs in the posterior CV (PCV, the CV analog in zebrafish), but rather arise from a subset of mesodermal precursor cells [193]. These cells are located in the ventral region of the PCV and begin to migrate dorsally as they specify into LECs. It is these groups of cells from which lymphatic sprouts begin to form [193]. These data highlight the continued debate on the origin of LECs and provide further support for the existing heterogeneity of LECs and plasticity in progenitor ECs.

### Non-venous-derived LECs

In 1910, Huntington and McClure were the first to propose a non-venous origin of LEC progenitor cells [194]. They showed that mesoderm-derived endothelial precursor cells specify into LECs independent of the venous endothelium. This theory was largely dismissed for the venous-derived theory until recently, where advancements in lineage-tracing studies provide further support for tissue-specific, non-venous origins of LECs. Using these models, non-venous progenitor cells were found to be derived from multiple cell types including mesenchyme [195–197], the paraxial mesoderm [192], hematopoietic cells [198], hemogenic progenitor cells [161, 172], and the skin capillary plexus [199]. The discovery of these non-venous lymphatic progenitor cells revealed a complexity in the diversity of cell progenitors and the LECs that make up the lymphatic circulation. Furthermore, the specific source of LEC progenitor cells seems to ultimately determine the organ- or tissue-specific lymphatic vessels that are formed.

Tissue-specific dependence of lymphatic development is best highlighted by the development of the dermal lymphatics. The dermal lymphatics are derived from both venous and non-venous progenitor cells, each contributing to different regions of the dermal lymphatic network. Deletion of *Prox1* in cells expressing EC-enriched *Tie2*, disrupts development of only the cervical and thoracic dermal lymphatic vessels [164]. These mice show significant subcutaneous edema; however, the growth and specification of the lumbar lymphatic vessels are unaffected, accounting for approximately 30% of LECs that were derived from non-venous origins (*Tie2*^*−*^ derived cells) [164]. LECs derived from the Tie2-expressing cells sprout from the developing lymph sacs via lymphangiogenesis. Conversely, the LECs derived from the *Tie2*-negative population appear as clusters which coalesce and grow into mature lymphatics through lymphvasculogenesis.

Similarly, cardiac and facial lymphatics are derived from both venous and non-venous cell origins. In cardiac development, venous-derived LECs form the dorsal lymphatic vessels. These LECs sprout from the sinus venosus to form the dorsal network [200]. The ventral lymphatic vessels, however, are derived from arterial sub-mesothelial cells that develop from the second heart field [200]. In zebrafish, facial lymphatics develops in three individual steps. The first set of facial lymphatic vessels develops through lymphangiogenesis and sprouts from the dorsal common CV. After the first facial lymphatics form, a subset of angioblasts that resides next to the ventral aorta begins to express PROX1 and proliferates to form the ventral aorta lymphangioblasts that connect to the already developed facial lymphatic vessels [195]. The formation of the ventral facial lymphatic vessels from angioblast progenitors may have evolved out of necessity for a non-venous progenitor pool. The ventral facial area of the developing zebrafish lacks venous circulation and likely necessitated a new source of LECs to be developed in order to have a mature lymphatic network.

Some studies have suggested that non-venous LECs may, in part, be derived from hematopoietic progenitor cells [201–203]. This theory is based on the evidence that hematopoietic cell lineage (*Vav*^+^) cells also express lymphatic-enriched genes, including *PDPN*, *LYVE1* and *VEGFR3*. A study using the cornea lymphangiogenesis model in irradiated mice, showed that bone marrow-derived LECs progenitor cells can exit the blood vasculature and infiltrate sites of lymphavasculogenesis [201]. The authors used EGFP-labeled bone marrow cells to trace immune cell infiltration into the cornea and determined that a subset of these EGFP-expressing cells also express lymphatic-enriched genes such as *Lyve1* and *Vegfr3* [201]. However, the potential of hematopoietic cells as a source of lymphatic progenitor cells continues to be debated.

Hemogenic ECs have also been identified as a potential source of LEC progenitor cells in the developing mesenteric lymphatics. Stanczuk et al*.* used lineage tracing of cells expressing cKit, which is highly enriched in hemogenic ECs, to assess their contribution in mesenteric lymphatic development [161]. Much like in the dermal lymphatics, mesenteric LECs are derived from both venous and non-venous cell populations. The venous population sprouts from the lymph sac at the mesenteric root and grows outward toward the intestine. These cells are PROX1^+^ and appear as early as E12.5. By E13, the LECs have grown sufficiently to form large collecting lymphatics. The non-venous cells, which express cKit, appear in clusters around the mesenteric vessels, and form a primitive plexus that joins the sprouting LECs from the lymph sac to form the mature mesenteric lymphatics. These cKit-expressing cells also express PROX1 and NRP-2, suggesting they are lymphatic progenitor cells. Collectively, these studies highlight the importance of understanding the cell and tissue heterogeneity of developing lymphatics in order to better understand the mechanisms that regulate lymphatic vascular development in both health and disease.

### Regulation of lymphatic valve formation

Collecting lymphatic vessels have intraluminal bicuspid valves to help prevent retrograde transport of lymph. Lymphatic valves are formed by a layer of LECs surrounding a fibronectin splice isoform EIIIA (FN-EIIIA)-, collagen IV- and laminin α5-rich core [204, 205]. The LECs that make up the valves are specified through a series of genetic programing that promotes LEC migration into the lumen of lymphatic vessels and organization into a functional valve [166, 204–207]. Mutations in these genetic programs are associated with human lymphedemas (Fig. 2, Table 1) [156, 166, 207].

Lymphatic valve formation occurs in four distinct stages: initiation, circularization, condensation and leaflet elongation [207]. Initiation occurs as a select few LECs upregulate lymphatic markers to specify the valve-forming region. This process is mainly driven by the transcription factor FOXC2 [206]. FOXC2 interaction with other transcription factors, including GATA2 and NFATc1, upregulates the expression of the lymphatic-enriched transcription factor PROX1 [206, 208]. High expression of PROX1 (PROX1^high^) specifies LECs into valve-forming cells. Deletion of *Foxc2* expression in mice reveals aberrant lymphatic valve formation leading to severe edema [209]. Activation of FOXC2 is mediated by shear stress in sites of disturbed flow, mainly due to vessel branching, and promotes valve formation [205]. Specifically, oscillatory shear stress upregulates FOXC2 activating the calcineurin/NFATc1 pathway to form lymphatic valves. FOXC2-dependent activation of NFATc1 is, in part, dependent on mechanosensitive gap junction channel proteins, Cx37 and Cx45 [205]. Loss of Cx37 or Cx43 in mice results in impaired lymphatic valve formation [210]. Further evidence for the role of shear stress in the developing lymphatic valve is seen in the development of lymphedema in *Piezo1*-deficient mice [211, 212]. PIEZO1 is a mechanosensitive ion channel whose activation has been linked to the development of lymphatic valves. Mice lacking *Piezo1* specifically in LECs fail to form lymphatic valves [211, 212]. Conversely, overexpression of *Piezo1* leads to increased number of lymphatic valves in the developing mice resulting from increased lymphatic valve gene expression [211]. Together, these findings highlight the importance of mechanical stress on LECs due to increased lymph flow to direct lymphatic valve development.

Similar to early LEC specification from progenitor cells, VEGF-C signaling plays an important role in the upregulation of PROX1 in the cells located in the valve-forming region [213]. VEGF-C-VEGFR3 signaling promotes the upregulation of PROX1 in valve-forming LECs through the activation of the transcriptional co-activators YAP and TAZ [213]. However, the mechanisms by which VEGF-C signaling promotes valve formation in restricted localized areas within the lymphatic vasculature remains unknown. Localized upregulation of PROX1 in valve-forming regions may be, in part, regulated by NOTCH signaling [214]. LEC-specific deletion of *Notch1* in mice results in the expansion of PROX1^high^ cells within the valve-forming region and misalignment of valve LECs in the developing valve leaflets [214]. NOTCH-1-dependent valve malformation is caused, in part, by decreased FN-EIIIA and integrin α9 in the valve-forming regions, revealing NOTCH-1 signaling as important, not only for valve LEC specification, but also for migration of these cells into a functional valve structure. BMP9-dependent signaling may also play an important role in lymphatic valve development. *Bmp9*^*−/−*^ mice have dilated collecting vessels and lymphatic valve malformations in the mesenteric lymphatics [215]. The collecting lymphatics from these mice maintain expression of LYVE1, which is downregulated during lymphatic maturation, suggesting that BMP9 signaling contributes to lymphatic maturation during late lymphatic development [215, 216].

As early valve-forming LECs are specified around the valve-forming regions (a.k.a. the circularization step), they begin to move and condense into a ring surrounding the lumen. At this time, cells begin to deposit the extracellular matrix that will form the core of the valve leaflets [204, 205]. This extracellular matrix is made up of FN-EIIIA, collagen IV and laminin α5 [204, 205]. Expression of integrin α9 by valve-forming LECs promotes cell migration through the newly deposited matrix to form the classic bicuspid valve [204]. Migration of these LECs is also driven by the activation of planar cell polarity pathways [217]. Specifically, activation of CELSR1 promotes LEC migration by destabilizing VE-cadherin interactions, while enhancing adherens junction assembly [217]. Careful regulation of all these pathways helps generate functional lymphatic valves, allowing for proper tissue fluid homeostasis in the developing embryo and into adulthood.

## Lymphedema and lymphatic malformations

Dysregulation of LEC specification leads to lymphedema and lymphatic vascular malformations, which can be debilitating and even fatal (Fig. 2, Table 1). We provide an overview of these disorders, what is known about their underlying genetic defects, and what treatments are currently available for affected patients.

### Lymphedema

The most frequent lymphatic anomaly is lymphedema (LE). LE is characterized by diffuse, localized or extended swelling due to inefficient uptake of interstitial fluid and reduced lymphatic drainage, mostly in the extremities [218]. There are two types of LE: primary LE, which are genetic disorders; and secondary LE, which develop due to extrinsic factors such as surgery or infection of lymphatic vessels.

### Primary congenital lymphedema

Inherited primary LE is classified as early-onset congenital LE (Nonne-Milroy lymphedema or Type I lymphedema, OMIM 153100), peripubertal (Meige disease or Type II lymphedema, OMIM 153200), or late-onset congenital LE (after 35 years of age, also named lymphedema tarda) [100, 219]. Around twenty gene mutations have been identified in different forms of LE [220]. The incidence of primary lymphedema is low, affecting 1 in 100,000 people worldwide [221]. Most of the genes involved encode for proteins in the VEGF-C/VEGFR3-signaling pathway [220].

The first mutations were discovered in *FLT4,* the gene encoding VEGFR3. Autosomal dominant and recessive missense mutations in the tyrosine kinase domain of this receptor leads to type I lymphedema [220, 222]. These mutations inhibit VEGFR3 phosphorylation and prevent its downstream signaling [222]. The homozygous *Flt4* knockout mice die around E9.5 due to irregular and unorganized vessels, edema and cardiovascular failure [223]. The *Chy* mice, a model of Type I lymphedema, have heterozygous inactivating mutations in *Vegfr3* and develop dysfunctional hypoplastic lymphatic vessels and swelling of the limbs [224]. Collagen and calcium-binding EGF domain-containing protein 1 (CCBE1) binds to the extracellular matrix to potentiate VEGF-C effects via VEGFR3 [225]. In humans, homozygous and heterozygous mutations that impair CCBE1 function cause the Hennekan lymphangiectasia-lymphedema syndrome (OMIM 235510), which includes generalized lymphatic anomalies, including LE, visceral lymphangiectasias and mental retardation [226]. In zebrafish, it was shown that CCBE1 is required for lymphangioblast budding and angiogenic sprouting from venous endothelium and its gene mutation leads to the full of fluid (*fof*) mutant phenotype [227]. PTPN14 is a tyrosine-phosphatase that regulates VEGFR3 activation after VEGF-C binding. A loss of function mutation of *PTPN14* leads to LE (OMIM 608911) due to VEGFR3-signaling hyperactivation [228].

Downstream of the VEGF-C-VEGFR3-signaling pathway, several transcription factors are activated and regulate numerous targets genes [220]. For example, truncated and missense mutations of *FOXC2* are found in patients with hereditary type II lymphedema (late-onset LE) [229]. *Foxc2* homozygous deletion in mice results in defective lymphatic patterning and arrested lymphatic valve development. *Foxc2* heterozygous and endothelial-specific deletion in mice leads to lymphatic hyperplasia and impaired valve function in collecting lymphatic vessels [209, 230]. A rare form of LE with variable onset, Hypotrichosis-Lymphedema-Telengiectasia (OMIM 607823), has been associated with recessive and dominant mutations in the transcription factor SOX18 [231, 232]. SOX18 regulates PROX1, a transcription factor essential for lymphangiogenesis which, in turn, positively regulates *FLT4* gene expression. The observed lymphatic defects may be due to competitive transcription factor binding [231].

GATA2 is another transcription factor that regulates PROX1 and FOXC2 expression. Loss-of-function mutations in *GATA2* have been reported in patients with primary LE and myelodysplasia (Emberger syndrome, OMIM 614038) [208]. GATA2 in expressed by ECs, hematopoietic stem and progenitor cells, and lymphatic valve-forming cells. *Gata2*-homozygous deletion in mice leads to embryonic lethally at mid-gestation due to anemia and reduced myeloid-erythroid progenitor cells. However, no vascular defects have been detected in this model, which may be due to redundancy among GATA family members [233, 234].

Impaired lymphatic valve development/function could contribute to LE, as mutations in *GJC1* encoding Cx43 and *GJC2* encoding Cx47, which are expressed in lymphatic valves, have been identified in some patients [235, 236]. The related mutations are amino acid substitutions that alter connexin functions. Substitutions of highly conserved amino acids in *GJC2* (Cx47) cause LE in all four extremities [235], whereas loss-of-function mutations cause Hypomyelinating leukodystrophy 2 (OMIM 608804), in which LE does not occur. The amino acid substitutions may have gain-of function effects, since *Gjc2* homozygous deletion in mice does not result in lymphatic defects [237]. Various mutations in *GJA1* (Cx43) are known to cause oculodentodigital dysplasia (OMIM 164200), and at least one has been linked to primary LE [236]. FOXC2 transcription factors also control the expression of several other proteins involved in lymphangiogenesis, such as Cx37, which is involved in lymphatic valve formation [210, 238]. Indeed, Cx37 expression is drastically reduced in mesenteric lymphatic vessels of mice lacking *Foxc2*, suggesting that *Gja4* (encodes Cx37) may be regulated by FOXC2 [210]. However, the mechanism underlying Cx37-, Cx43- or Cx47-related LE is still unclear and could be related to abnormal lymphatic vessel and/or valve development, or defective cell–cell communication in lymphatic vessels leading to impaired coordination of pulsatile lymphatic flow [239]. Interestingly, Cx37 is a potent inhibitor of cell cycle progression, and it is therefore possible that Cx37 plays a similar cell cycle arrest role via p27 to enable specification toward lymphatic EC fates [240].

### Lymphatic malformations

Lymphatic malformations (LM) consist of masses of abnormal dilated lymphatic channels not connected to the lymphatic system but filled with fluid, most commonly located in the head and neck [100, 220]. LMs are sporadic and their etiopathogenesis is unknown. LM can be part of a syndrome, such as Turner syndrome (due to monosomy X), or overgrowth syndrome, such as Klippel-Trenaunay syndrome (capillary-lymphatico-venous malformation, OMIM 149000) that is caused by mutations in the PI3K/AKT pathway [220].

## Clinical treatments for lymphatic vascular malformations

The current therapy for lymphedema consists of decongestive physiotherapy to reduce edema and maintain the health of the skin and surrounding structures. Manual lymphatic drainage, as well as skin care and exercise, and the use of compression bandages are the gold standards. Occasionally surgery is performed [241, 242]. These treatments can only result in symptomatic improvements, but they do not cure the underlying dysfunction. Medical treatment of complicated LM with Sirolimus has showed substantial clinical benefits, though further research is needed to determine the efficacy of this medication for diverse subsets of lymphatic malformations [242, 243]. Furthermore, preclinical studies in mice have shown that Sirolimus treatment combined with anti-VEGF-C therapy can promote the regression of LM in *Pik3ca*-mutant model [244].

## Summary

EC specification is a critical step in vascular development. Perturbations in the signaling pathways that determine blood and lymphatic EC identity result in vascular malformations. A combination of factors, including growth factor signaling, transcriptional regulation, and mechanotransduction, determine EC fate in the developing embryo. Furthermore, there is a growing appreciation for the role of cell cycle regulation in EC fate determination. Impairment of cell cycle control results in aberrant EC growth and fate determination that may ultimately lead to vascular malformations. Further research is still needed to elucidate the mechanisms of EC specification, and how their dysregulation leads to vascular malformations. Gaining further insights will help to improve treatment for such disorders.
